# The necessity of CT scans on pediatric carotid injury after blunt trauma – An analysis of the traumaregister DGU^®^

**DOI:** 10.1007/s00068-025-03049-5

**Published:** 2026-01-13

**Authors:** Lars Becker, Lukas Krüger, Maximilian Wolf, Katharina Alfen, Jens Theysohn, Rolf Lefering, Marcel Dudda, Oliver Kamp, Committee on Emergency Medicine, Intensive Care and Trauma Management (Sektion NIS) of the German Trauma Society (DGU), Germany

**Affiliations:** 1 Department of Trauma Surgery, Hand and Reconstructive Surgery, University Hospital Essen, 45147 Essen, Germany; 2 Department of Orthopedics and Trauma Surgery, Evangelisches Klinikum Gelsenkirchen, 45879 Gelsenkirchen, Germany; 3 Department of Paediatrics I, Neonatology, Pediatric Intensive Care Medicine and Pediatric Neurology, University Hospital Essen, 45147 Essen, Germany; 4 Department for Diagnostic and Interventional Radiology and Nuclear Medicine, University Hospital Bergmannsheil Bochum, 44789 Bochum, Germany; 5 Institute for Research in Operative Medicine (IFOM), Witten/Herdecke University, 51109 Cologne, Germany; 6 Department of Orthopaedics and Trauma Surgery, BG-Klinikum Duisburg, 47249 Duisburg, Germany

**Keywords:** Blunt carotid injury, Pediatrics, TraumaRegister DGU^®^, CT, Radiation risks

## Abstract

**Purpose:**

Blunt carotid injuries (BCI) in pediatric trauma patients are rare. Using data from the TraumaRegister DGU^®^^,^ this study aims to identify screening parameters and calculate the prevalence of pediatric BCI. By proposing potential risk factors for a BCI, this research seeks to reduce unnecessary radiation exposure in pediatric trauma cases. These findings may enhance understanding of pediatric BCI and highlight the necessity of cautious diagnostic approaches that balance clinical needs with radiation risks.

**Methods:**

The TraumaRegister DGU^®^ is a multicenter database established in 1993 to document the treatment of severely injured patients from initial injury to hospital discharge. Data are collected in four phases: demographics, injury patterns, treatments, and outcomes. Almost 700 hospitals, primarily from Germany, contribute to the registry annually. Statistical analysis was conducted using SPSS. For analysis, the dataset was divided into two groups: trauma patients diagnosed with BCI and trauma patients without BCI. The complete dataset from the TraumaRegister DGU^®^ for 2006–2020 was screened for relevant cases. The dataset was limited to patients between 0 and 15 years old.

**Results:**

Out of 9070 severely injured pediatric trauma patients analysed, 50 cases of pediatric BCI were identified, representing a prevalence of 0.6%. Patients with BCI presented with higher injury severity scores (ISS), lower Glasgow Coma Scale (GCS) scores, and a greater prevalence of head injuries, as well as thoracic, abdominal, and extremity injuries. These patients also experienced higher in-hospital mortality rates (34%) and required more frequent blood transfusions. Full-body CT scans were more commonly performed in patients with BCI.

**Conclusion:**

This study highlights the rarity and severity of BCI in pediatric trauma patients, with a prevalence of 0.6%. Significant risk factors for a BCI include high injury severity, head trauma, neurological deficits, and pre-hospital hypotension. The findings emphasise the importance of early diagnosis and targeted diagnostic strategies to balance the need for prompt intervention with reducing unnecessary radiation exposure.

## Introduction

Blunt carotid injuries (BCI) in pediatric trauma patients are exceedingly rare. As a narrative review article, our latest literature research has shown an overall prevalence of 0.03% to 0.5% in different registry studies from the US and Germany published in the last 25 years [1]. The delayed diagnosis of BCI is common, mainly due to the late onset of symptoms, which increases the risk of stroke and long-term complications. Additional complications include Horner’s syndrome, pseudaneurysm formation and various vascular issues, all of which can result in severe outcomes such as permanent disability or death. Early recognition and prompt treatment are crucial for improving prognosis and minimising long-term damage. Computed tomography angiography (CTA) is currently considered the “gold standard” for detecting BCI [2, 3]. Up to 16% of pediatric patients with blunt trauma underwent imaging procedures to identify BCI, with the majority, around 64% to 71% undergoing CTA [1–4]. So far, there is only one relevant clinical score, the McGovern score, which shows a sensitivity of 81% and specificity of 71.3% in BCI prediction [3]. However, concerns have been raised regarding the potential long-term effects of radiation exposure, particularly in pediatric patients. Different studies suggest that even minimal radiation exposure may increase the risk of pediatric cancer [5, 6]. As there are no guidelines for routine diagnostic work-up in pediatric patients to identify BCI, we need to establish a diagnostic standard because early detection is critical for preventing serious complications, such as ischemic stroke and permanent neurological damage. The variable presentation and delayed symptom onset in children often lead to missed or late diagnoses, increasing the risk of adverse outcomes. Approximately 76% of carotid and 67% of vertebral arteries were re-evaluated using arteriography 7 to 10 days post-injury [7]. A standardised diagnostic protocol would ensure timely identification, appropriate imaging, and early intervention, ultimately improving prognosis, reducing long-term disabilities and optimising treatment strategies for affected children. Therefore, looking for possible screening parameters and risk factors for pediatric BCI is necessary.

This retrospective study aims to identify potential screening parameters and risk factors for pediatric BCI and reduce unnecessary radiation exposure. Furthermore, it seeks to investigate the frequency of CT scans performed for BCI diagnosis in pediatric trauma cases and assess the number of scans that yield negative results.

## Materials and methods

The TraumaRegister DGU^®^, established by the German Trauma Society (Deutsche Gesellschaft für Unfallchirurgie, DGU) in 1993, is a multicenter database designed for pseudonymized and standardised documentation of severely injured patients.

Data are collected prospectively across four distinct phases: (A) the pre-hospital phase, (B) emergency room and initial surgery, (C) intensive care unit (ICU) management, and (D) discharge. The documentation encompasses comprehensive details on demographics, injury patterns, comorbidities, pre- and in-hospital management, ICU course and relevant laboratory findings, including transfusion data and outcomes for each patient. Inclusion criteria consist of admission to a hospital through an emergency room with subsequent ICU care or arrival at the hospital with vital signs, but death before ICU admission.

The Academy for Trauma Surgery (AUC - Akademie der Unfallchirurgie GmbH), affiliated with the German Trauma Society, provides the infrastructure for documentation, data management, and analysis. Scientific oversight is managed by the Committee on Emergency Medicine, Intensive Care, and Trauma Management (Sektion NIS) of the German Trauma Society. Participating hospitals submit pseudonymized data into a central database using a web-based application. Scientific data analyses are subject to approval through a peer review process per the publication guidelines of the TraumaRegister DGU^®^.

The majority of participating hospitals are located in Germany. However, many hospitals from other countries contribute data, including Austria, Belgium, China, Finland, Luxembourg, Slovenia, Switzerland, the Netherlands, and the United Arab Emirates. Approximately 31,000 cases from nearly 700 hospitals are entered into the database annually.

Participation in the TraumaRegister DGU^®^ is voluntary. However, hospitals affiliated with the TraumaNetzwerk DGU^®^ are required to enter at least a basic dataset for quality assurance purposes. The complete dataset from the TraumaRegister DGU^®^ for 2006–2020 was screened for relevant cases. The following Abbreviated Injury Scale (AIS) codes were considered as BCI: 1228xx, 3202xx, 3204xx, 3210xx (AIS 2005 – Update 2008, AAAM Association for the Advancement of Automotive Medicine, Barrington, IL, USA). The dataset was delimited to Patients in the age range of 0 to 15 years. We excluded patients treated outside Europe to eliminate the influence of different medical systems. Only primary admitted children were considered; patients transferred from other hospitals were excluded. Adult patients with an age of ≥ 16 years or unknown age were also excluded from the analysis. Finally, all patients with minor or moderate injuries (worst injury according to the maximum Abbreviated Injury Scale (MAIS) ≤ 2) were excluded.

We reviewed all the excluded patients with an MAIS ≤ 2 to prevent falsifying the statistical results. No BCI patients were found in this excluded group.

This study adheres to the current publication guidelines of the TraumaRegister DGU^®^ and is registered under the TraumaRegister DGU^®^ project ID 2020-062.

### Statistics

Statistical evaluation was conducted using SPSS (Version 29.0, IBM Inc., Armonk, NY, USA). Categorical variables were presented as numbers with percentages, and metric data were presented as the mean with standard deviation (SD) plus the median. For selected findings, a 95% confidence interval was provided (CI_95_). Results were presented for patients with and without BCI. Differences were evaluated with the chi-squared test and the Mann-Whitney U-test, respectively. The significance level was set at 5% (*p* < 0.05). Missing values were not imputed; only real measurements were reported.

## Results

After applying all inclusion and exclusion criteria, 9,070 patients remained for further analysis. Within this dataset, we identified 50 pediatric blunt carotid injury (BCI) cases, resulting in an overall prevalence of 0.55% (CI_95_ 0.41–0.73). In the subgroup of polytrauma patients (*n* = 1,402), defined as ISS ≥ 16 [8], we identified 25 cases with BCI, which resulted in a prevalence rate of 1.8% (CI_95_ 1.1–2.5). The average age of the patients was 8.9 (SD 4.8) years with a median of 10 years. In comparison, the age of the BCI patients was higher, with a mean of 10.3 (SD 4.8) and a median of 12 years (*p* = 0.034). The age distribution is shown in Table 1. 63.2% of all patients were male, 36.8% female. In the group of BCI patients, 56.0% were male, 44.0% were female (*p* = 0.29).

**Table 1:** Age distribution

			Without BCI	With BCI
Age in groups	0–1	n	848	3
		%	9.4%	6.0%
	2–5	n	1663	8
		%	18.4%	16.0%
	6–9	n	1842	8
		%	20.4%	16.0%
	10–13	n	2446	11
		%	27.1%	22.0%
	14–15	n	2221	20
		%	24.6%	40.0%
Total		n	9020	50

BCI = blunt carotid injury, n = number

**Fig. 1:**
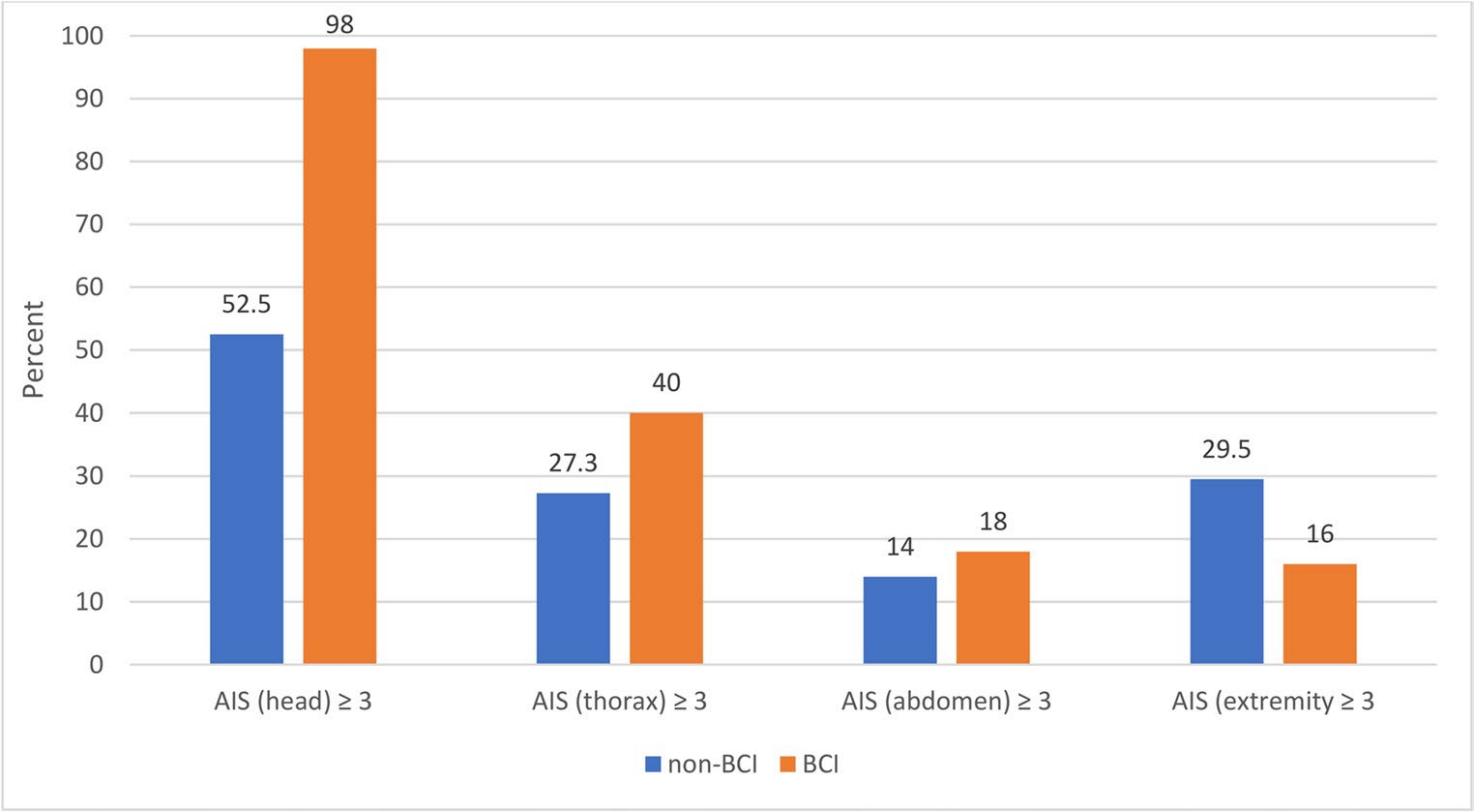
Prevalence of serious injuries (Abbreviated Injury Scale AIS ≥ 3) in different body regions in percent, in patients with and without blunt carotid injuries (BCI)

The mean number of body regions with a serious injury (AIS ≥ 3) in the group of BCI patients was 2.8, in the group of non-BCI patients, 1.8 (*p* < 0.001). A clear dependence between the injured body region and BCI was observed for severe head injury (AIS ≥ 3), other than the BCI: 98.0% (49 of 50) versus 52.5% (*p* < 0.001) without BCI. BCI patients also had more frequent thoracic and abdominal injuries, but not for extremities (Fig. 1).

The mean Injury Severity Score (ISS) in trauma patients without BCI was 19.5. In the group of patients with BCI, the mean ISS was much higher, with 35.7 (*p* < 0.001). All results are shown in detail in Table 2. Prehospital Glasgow Coma Scale (GCS) was recorded in 8,029 cases of trauma patients without BCI and in 46 cases of patients with BCI. Unconsciousness (GCS ≤ 8) was found in 67.4% of BCI patients but only in 25.4% of non-BCI patients (*p* < 0.001). The Eppendorf-Cologne Scale (ECS) is a combination of pupil size (0–2 points), light reaction (0–3 points) and motor response (0–3 points). The ECS was documented in 6,052 cases, 33 BCI cases. ECS was normal (defined as ECS = 0) in 56.6% of non-BCI cases but only in 18.2% of BCI cases.

**Table 2:** Comparison of patients with and without BCI

	Without BCI *n* = 9020	With BCI *n* = 50	*p*-value
AIS (head) ≥ 3(excluding the code for BCI)	4735 (52.5%)	49 (98.0%)	*p* < 0.001
AIS (thorax) ≥ 3	2464 (27.3%)	20 (40.0%)	*p* = 0.055
AIS (abdomen) ≥ 3	1259 (14.0%)	9 (18.0%)	*p* = 0.412
AIS (extremities) ≥ 3	2658 (29.5%)	8 (16.0%)	*p* = 0.052
Injury Severity Score (ISS) *	19.5 (11.5) median 17	35.7 (13.3) median 17	*p* < 0.001
Unconscious (GCS ≤ 8)	2037 (25.4%)	31 (67.4%)	*p* < 0.001
Eppendorf Cologne Scale (ECS) (pupils and motor response; 0 = normal)			*p* < 0.001
0	3405 (56.6%)	6 (18.2%)	
1	1002 (16.6%)	3 (9.1%)	
2	373 (6.2%)	6 (18.2%)	
3	379 (6.3%)	1 (3.0%)	
4	199 (3.3%)	3 (9.1%)	
5	126 (2.1%)	1 (3.0%)	
6	111 (1.8%)	3 (9.1%)	
7	84 (1.4%)	1 (3.0%)	
8	340 (5.6%)	9 (27.3%)	
Syst. BP ≤ 90 mmHg pre-hospital	1135 (16.6%)	14 (35.9%)	*p* = 0.001
Syst. BP ≤ 90 mmHg on admission	1071 (13.4%)	9 (20.9%)	*p* = 0.174
Blood transfusion	741 (8.3%)	15 (31.3%)	*p* < 0.001
Coagulopathy	1098 (15.4%)	19 (44.2%)	*p* < 0.001
Hospital mortality	666 (7.4%)	17 (34.0%)	*p* < 0.001
Deaths within 24 h	484 (72.7%)	15 (88.2%)	*p* < 0.001
Hospital stay in days *	11.1 (13.2) median 8	14.4 (19.6) median 6	
Days in ICU *	4.6 (8.0) median 2	11.3 (18.6) median 3	
Time from injury to hospital admission, in minutes *	61.2 (31.2) median 56	70.2 (34.2) median 63	*p* = 0.097
Hospital level of care			*p = 0.324*
Level 1	6279 (69.6%)	39 (78.0%)	
Level 2	2190 (24.3%)	10 (20.0%)	
Level 3	551 (6.1%)	1 (2.0%)	
Helicopter transport to hospital	2402 (27.7%)	17 (35.4%)	*p* = 0.257
Initial CT of head and neck	7098 (78.7%)	48 (96.0%)	*p* < 0.001
Whole-body CT	5087 (57.9%)	42 (85.7%)	*p* < 0.001
Time to CT in minutes *	23.4 (18.5) median 19	19.6 (11.6) median 17	*P* = 0.224

* mean (SD) median

BCI = blunt carotid injury, n = number, AIS = Abbreviated Injury Scale, SD = standard deviation, GCS = Glasgow Coma Scale, ECS = Eppendorf-Cologne Scale, syst. BP = systolic blood pressure, mmHG = millimeter of mercury, ICU = intensive care unit, CT = computed tomography

Pre-hospital systolic blood pressure (syst. BP) was available in 6,885 cases. A shock (syst. BP ≤ 90 mmHg) was found in 14 BCI patients (35.9%) versus 16.6% in non-BCI patients (*p* = 0.001).

Slightly more BCI patients were initially brought to a (supraregional) level 1 trauma centre and transported to the hospital with a helicopter, but these differences were not significant. The injury mechanisms are presented in Table 3. BCI was found more frequently in car passengers and less regularly in low falls.

**Table 3:** Distribution of injury mechanisms in patients with and without BCI

	Without BCI	With BCI
Traffic: car	1087	11
	12.3%	22.4%
Traffik: motorcycle	525	4
	5.9%	8.2%
Traffic: bicycle	1311	5
	14.8%	10.2%
Traffic: pedestrian	1694	10
	19.1%	20.4%
High fall (> 3 m)	1477	8
	16.7%	16.3%
Low fall (< 3 m)	1284	3
	14.5%	6.1%
Other	1480	8
	16.7%	16.3%

BCI = blunt carotid injury, m = meter

The mean time between the injury and hospital admission was 61 min in BCI cases and 70 min in non-BCI patients (*p* = 0.097).

The blood transfusion rate in the hospital was significantly higher in the BCI group (*p* < 0.001). Coagulopathy on admission was defined as one of the following: Quick’s value ≤ 60%, partial thromboplastin time (PTT) ≥ 40 s., or International Normalised Ratio (INR) ≥ 1.4. The rate of coagulopathy was three times higher in BCI patients (44.2% versus 15.4%, *p* < 0.001). Mortality in patients with BCI was 34% (17 of 50) compared to non-BCI patients with 7.4% (*p* < 0.001). Length of stay in the ICU and in the hospital was also prolonged in BCI patients.

Initial diagnostic workup in the emergency room included cranial CT (selective or as part of a whole-body CT), which was performed in 48 of 50 BCI patients (96.0%) but only in 78.7% of non-BCI patients (*p* < 0.001). Also, whole-body CT was performed more frequently in BCI patients. The average time between hospital admission and the first CT scan was 19.6 min in the group of BCI patients vs. 23.4 min in the non-BCI group (*p* = 0.224).

## Discussion

This study investigated the prevalence of blunt carotid injury (BCI) in pediatric trauma patients, along with potential screening parameters and risk factors. The overall prevalence of BCI in a cohort of severely injured children in the TraumaRegister DGU^®^ between 2006 and 2020 was 0.55%, highlighting the rarity of such injuries in pediatric trauma cases. A literature review from our group published in 2024 has shown an overall prevalence between 0.03 and 0.5% of confirmed BCI cases due to pediatric blunt trauma, which almost reflects our result in this work [1, 2, 9–15]. This study revealed that all BCI patients had a MAIS ≥ 3, indicating severe injuries. This highlights the severity of these injuries and the need for comprehensive evaluation in suspected cases.

Head injuries are a significant differentiator between trauma patients with and without BCI. Nearly all BCI patients had an AIS (head) score ≥ 3 for head injury, in addition to the AIS codes concerning the BCI, emphasising the importance of evaluating head trauma as a potential indicator for BCI. BCI patients exhibited a higher Injury Severity Score (ISS) compared to non-BCI patients, indicating a greater overall trauma burden. There is no clinical score for BCI that can be assessed using the AIS or ISS. There are five clinical screening scores for BCI: Memphis criteria [16], Denver [17], EAST [18], Utah [19] and the McGovern score [3]. A study conducted at the University of Missouri-Columbia revealed that the modified Memphis criteria misclassified 28.6% of pediatric trauma cases. Similarly, other scoring systems, including the Denver, EAST, and Utah scores, misclassified 28.6%, 33.3%, and 47.6% of cases, respectively. Only the McGovern score demonstrated a sensitivity of 81% and a specificity of 71.3% for accurately detecting BCI [3]. The clinical McGovern score uses six elements that were identified as risk factors for BCI: Glasgow Coma Scale (GCS) < 8, focal neurological deficit, carotid canal fracture, petrous temporal bone fracture, cerebral infarction on CT, and a motor vehicle accident as a mechanism of injury (MOI) [3]. We also assessed GCS and the Eppendorf Colone Scale (ECS) scores as potential screening parameters. BCI patients were more likely to present with lower GCS and higher ECS scores, suggesting more significant neurological deficits. Also, a vehicle accident as MOI could be confirmed as a potential risk factor for BCI by our database; 60% of all BCI patients were involved in a roadside accident. It was impossible to check the other risk factors because they were not recorded in our database. Furthermore, pre-hospital hypotension was found to be associated with BCI, and BCI patients were more likely to require red blood cell transfusions during initial treatment, further reflecting the severity of their trauma. Shock rates were probably lower on hospital admission due to volume therapy during initial per-hospital treatment. Coagulopathy was also more prevalent in BCI patients, indicating the complex nature of their injuries and the potential need for targeted management. The study demonstrated a higher mortality rate among BCI patients, with a considerable proportion of 30% (*n* = 15/50) dying within the first 24 h of admission. This highlights the critical need for early detection and intervention to improve outcomes for these patients. In terms of diagnostics, the study revealed that BCI patients were significantly more likely to undergo an initial CT scan in the emergency room, highlighting the clinical suspicion of BCI in these cases. Furthermore, more BCI patients received whole-body CT scans compared to non-BCI trauma patients, which may be indicative of a higher degree of clinical vigilance for these patients. Another study on the TraumaRegister DGU^®^ from 2019 has shown that in a pediatric control and BCI group, children with BCI underwent immediate head/neck CT in 85.3% vs. 94.4%, or whole-body CT in 64.6% vs. 86.1% [9]. However, based on our dataset, it remains speculative whether the decision to do a CT scan was ultimately due to the severity of the injury or the suspicion of BCI.

In knowledge, that the median volume CT dose index on a non-contrast head CT is about 33 milligray (mGy) [20], a CT of the skull or facial bones needs about 27–37 mGy, a scan of petrous bones needs about 42–67 mGy depending on the age group [21], and a computed tomography angiography needs at least 138 mGy [22], we should keep the radiation risk on pediatric patients in mind. Regarding radiation exposure and the risk of cancer, many publications have already proven the correlation between radiation exposure and the possibility of the appearance of cancer [5, 23, 24]. As we know, a cumulative radiation dose of about 50 mGy is sufficient to triple the risk of leukaemia, and a dose of about 60 mGy to triple the risk of brain cancer [23]; consideration should be given to whether the number of CT examinations can be reduced by identifying risk factors.

### Limitations of the study

The retrospective design of our study limits the extent of conclusions that can be drawn from the dataset. The dataset does not provide detailed information on the morphology, anatomical localisation, or type of carotid injuries, as it only documents the presence of the injury. Additionally, the dataset does not specify the methods used to screen carotid injuries- CT, MRI, ultrasound, or angiography.

Furthermore, the dataset does not include information on the radiation doses applied, making it impossible to assess the exact exposure levels and their potential impact on patient safety. Additionally, since accident history is not reported in all cases, it remains unclear if some injuries resulted from iatrogenic causes or shrapnel trauma.

Further research is needed to address these limitations. Ideally, prospective randomised studies with a data pool specifically designed to investigate pediatric carotid injuries resulting from blunt trauma would provide more comprehensive insights. Such studies should focus on detailed injury characteristics, diagnostic methods, and radiation exposure to enhance our understanding and improve clinical management of these injuries.

## Conclusion

This study provides critical insights into the characteristics, risk factors, and outcomes associated with blunt carotid injury (BCI) in pediatric trauma patients. With an overall prevalence of 0.6%, BCI remains a rare but severe complication, frequently linked to significant trauma in all detected cases. The strong association between BCI and head injuries highlights the importance of evaluating severe head trauma as a key indicator for BCI. Furthermore, BCI patients exhibited markedly higher Injury Severity Scores (ISS) and neurological impairment scores compared to non-BCI trauma patients.

The study also identified pre-hospital hypotension, coagulopathy and a higher need for blood transfusions as significant markers of the injury’s severity and systemic impact. Mortality among BCI patients was strikingly high, highlighting the urgency of timely diagnosis and intervention.

Given the established risks of radiation-related malignancies, there is a pressing need to refine risk stratification criteria to limit unnecessary imaging without compromising diagnostic accuracy. Since a BCI is obviously very unlikely in an otherwise non severely injured child, performing a CT scan solely to rule out a BCI without any signs of an overall seriously injured child should be strictly avoided and cannot be recommended.

This study corroborates the importance of established clinical screening tools like the McGovern score while emphasising the potential utility of integrating parameters such as GCS, ECS, ISS, AIS (head), pre-hospital systolic blood pressure and coagulopathy to improve the score further and increase its accuracy. These findings advocate for a balanced approach that prioritises early detection and treatment of BCI while safeguarding pediatric patients from the long-term risks associated with unnecessary diagnostic radiation.

## Data Availability

No datasets were generated or analysed during the current study.
